# The Multidimensional Assessment of Interoceptive Awareness, Version 2 (MAIA-2)

**DOI:** 10.1371/journal.pone.0208034

**Published:** 2018-12-04

**Authors:** Wolf E. Mehling, Michael Acree, Anita Stewart, Jonathan Silas, Alexander Jones

**Affiliations:** 1 University of California San Francisco, Department of Family and Community Medicine, San Francisco, CA, United States of America; 2 University of California San Francisco, Osher Center for Integrative Medicine, San Francisco, CA, United States of America; 3 University of California San Francisco, Institute for Health and Aging, San Francisco, CA, United States of America; 4 Middlesex University London, School of Science & Technology, Department of Psychology, London, United Kingdom; University G d'Annunzio, ITALY

## Abstract

Interoception, the process by which the nervous system senses, interprets, and integrates signals originating from within the body, has become major research topic for mental health and in particular for mind-body interventions. Interoceptive awareness here is defined as the conscious level of interoception with its multiple dimensions potentially accessible to self-report. The Multidimensional Assessment of Interoceptive Awareness (MAIA) is an 8-scale state-trait questionnaire with 32 items to measure multiple dimensions of interoception by self-report and was published in November 2012. Its numerous applications in English and other languages revealed low internal consistency reliability for two of its scales. This study’s objective was to improve these scales and the psychometrics of the MAIA by adding three new items to each of the two scales and evaluate these in a new sample. Data were collected within a larger project that took place as part of the Live Science residency programme at the Science Museum London, UK, where visitors to the museum (*N* = 1,090) completed the MAIA and the six additional items. Based on exploratory factor analysis in one-half of the adult participants and Cronbach alphas, we discarded one and included five of the six additional items into a Version 2 of the MAIA and conducted confirmatory factor analysis in the other half of the participants. The 8-factor model of the resulting 37-item MAIA-2 was confirmed with appropriate fit indices (RMSEA = 0.055 [95% CI 0.052–0.058]; SRMR = 0.064) and improved internal consistency reliability. The MAIA-2 is public domain and available (www.osher.ucsf.edu/maia) for interoception research and the evaluation of clinical mind-body interventions.

## Introduction

The Multidimensional Assessment of Interoceptive Awareness (MAIA)[1] is a 32-item state-trait questionnaire to measure multiple dimensions of interoception by self-report. Since its publication in November 2012, the MAIA has been translated into 20 other languages and used in numerous studies worldwide (see website www.osher.ucsf.edu/maia). Nine foreign-language validation studies have been completed, which generally confirm the original factor structure but also reveal important shortcomings.

Interest in interoception with research conducted in a wide variety of disciplines has grown in recent years, and its terminology has further evolved.[2] In particular, the term ‘interoceptive awareness’ and its proper operationalization have been disputed with various, and often diverging, views.[3] Most recently, Khalsa and colleagues published a white paper intended to settle some of these taxonomy issues.[4] The way we have conceptualized the term ‘interoceptive awareness’ during the development of the MAIA is comparable to what recently has been termed ‘interoceptive *sensibility*’.[5] Interoceptive awareness is a relatively broad term with ample space for defining, conceptualizing and operationalizing multiple aspects and dimensions as elements of the conscious processes of interoception that may be accessible to self-report.

A key element of interoception that has been operationalized and is widely used in interoception research is the concept of interoceptive accuracy. Most recently, interoceptive accuracy has also been labeled interoceptive *sensitivity*, not to be confused with sensibility.[6] It has now been shown, in numerous studies, that objectively measured interoceptive accuracy (or sensitivity) does not clearly correlate with subjective interoceptive self-report measures.[6–10] Initially, and in previous research, the term “interoceptive awareness” has unfortunately and frequently been associated with interoceptive accuracy.[7] More recently however, “interoceptive awareness” has been used in a few studies to indicate a ‘metacognitive awareness’ of interoceptive accuracy, operationalized using self-assessed confidence ratings in one’s ability to detect their own heart beat without feeling for a pulse.[11] That use of ‘awareness’ has been critiqued as reductionist and as missing the richness of the phenomenology of one’s inner experience.[12] Interoception has been defined as the process by which the nervous system senses, interprets, and integrates signals originating from within the body, providing a moment-by-moment mapping of the body's internal landscape across *conscious* and *unconscious* levels.[2] Interoceptive awareness here is defined as the conscious level of interoception with its multiple dimensions potentially accessible to self-report.

Clinically, an increased attentional focus on physical sensations has commonly been associated with anxiety, hypervigilance, somatization and hypochondriasis.[13] This style of interoceptive bodily awareness is viewed as maladaptive and potentially unhealthy. However, with mindfulness, mind-body approaches and the wide realm of bodywork techniques entering into the field of interest for scientific study, a very different style of interoceptive bodily awareness—mindful rather than anxiety-driven—has become a topic of interest for a growing group of researchers from neuroscience to integrative medicine and religious studies.[14, 15] Arriving from such disparate viewpoints, in recent years this field has experienced an exciting exchange and confluence of ideas and concepts.[3, 14, 16]

However, as robust research depends on valid measurements, little progress has been made regarding reliable objective measures. The validity of the most commonly used objective measures for interoceptive accuracy, the heart beat detection and counting tasks, has been questioned.[9, 17] Furthermore, recent studies question whether measuring heart beat detection accuracy is a measure of external criterion validity for what is actually of clinical importance in regards to variations of interoceptive skills.[18, 19] Using perturbances of autonomic heartbeat function and measuring the related interoceptive ability may aid in clarifying basic science questions regarding interoception[20] but may be viewed as a rather artificial context limiting its capability to capture the richness of interoceptive phenomenology.[12, 21] Regarding self-report measures of interoception, the legacy measures are the Body Perception Questionnaire (BPQ),[22] the Body Awareness Questionnaire (BAQ),[23] and the older brief Private Body Consciousness Scale (PPCS),[24] which are either limited to proxy symptoms for anxiety or otherwise lacking in capturing regulatory aspects of interoception.[25] In addition to the Body Responsiveness Scale (BRS)[26] and the Scale of Body Connection (SBC),[27] the MAIA is—despite its shortcomings—currently still the most widely used self-report measure of interoceptive bodily awareness.

The MAIA consists of eight scales corresponding to its 8-factor structure.[1] These are labeled Noticing, Not-Distracting, Not-Worrying, Attention Regulation, Emotional Awareness, Self-Regulation, Body Listening, and Trust. Non-Distracting indicates the tendency to ignore or distract oneself from sensations of pain or discomfort. Not-Worrying indicates emotional distress or worry with sensations of pain or discomfort. The MAIA is a self-report measure. There are limitations inherent in the self-report approach to assessing any psychological trait that include, but are not limited to response bias, state dependencies and social desirability. However, there is no well-defined objective measure for the dimensions of interoceptive bodily awareness (see an in-depth discussion of this limitation in [1], page 20, and [28], page 8). In several studies of the original English version and its translations, Cronbach alphas for these two scales were below the published version and less than optimal. This was in part explained by two characteristics of these scales: first, both had reversely scored ‘negative’ items, whereas all other scales had only positively scored items. Second, both scales consisted of only three items, and Cronbach alpha is sensitive to the number of scale items.[29] Despite their low internal consistency reliability, both scales have been valuable in discriminating between groups expected to differ due to known characteristics[30, 31] and in mediating the benefits of a clinical intervention.[32] Therefore, we have attempted to improve these two scales. Here we report results for a modification of the MAIA to add items to these two scales and conduct new psychometric assessments. The items of the other six scales remained unchanged.

## Methods

### Participants

Participants in this study were a convenience sample of visitors of the Live Science residency project at the Science Museum of London, UK. Participants were between 18 to 69 years old and able to comprehend English. We required a sample of at least 500 participants to feel confident about conducting appropriate factor analyses.

### Setting

Data collection for this study was part of a larger project that took place as part of the Live Science residency programme run at the Science Museum London, UK. In a dedicated space in a gallery within the museum, visitors to the museum could complete questionnaires on tablet devices and take part in experimental research on dedicated desktop computers. The overall project aimed at examining the relationship between cognitive and perceptual processes about the self and others. In addition to completing the MAIA, participants were also invited to take part in three reaction time-based experiments investigating tactile attention, mental rotation of bodies and action perception. The participants always completed the MAIA questionnaire first, and this task was completely independent from the other tasks. Participants were all above the age of 7 years, participation was voluntary and on an opportunistic basis. The residency programme ran over a period of six weeks where researchers were present three days a week during museum opening times to collect data on a voluntary basis from visitors able to provide informed consent (for those under the age of 18, consent was obtained from a legal guardian). Data from the experimental tasks and those under the age of 18 will be published separately. The study was approved by the Middlesex University Psychology ethics board (Project ID: 1846).

### Instruments

As the purpose of this publication is to develop an updated version of the MAIA with improved internal consistency for two of the eight MAIA scales, we used the original 32-item MAIA^1^ and six additional new items, three for each of the two problem scales mentioned above. The new items were derived by a team discussion among the same experts that created the original items, in part drawing from the original item pool collected with focus groups.[33] First, for Non-Distracting the new items were (1) *I try to ignore pain* (R); (2) *I push feelings of discomfort away by focusing on something else* (R); (3) *When I feel unpleasant body sensations*, *I occupy myself with something else so I don’t have to feel them* (R). R indicates reverse scoring so that higher values go along with stronger interoceptive skills. Second, for Not-Worrying the new items were (1) *When I feel an unpleasant body sensations I just let it go by*; (2*) I can stay calm and not worry when I have feelings of discomfort or pain*; and (3) *When I am in discomfort or pain I can’t get it out of my mind* (R). Results obtained with other questionnaires and behavioral tests applied in this study will be published separately.

As mentioned above, one possible cause for the low Cronbach alphas of the two scales was the reverse scoring. It is our view—and that of the original focus group participants[1]—that distraction and worrying are essential but inverse dimensions of interoceptive awareness, so that high scores go along with low interoceptive awareness. As all scales in a multidimensional questionnaire preferably would have the same direction for the overall construct (high scores should consistently indicate high interoceptive awareness), our focus group participants had created items that are consistent with distraction and worry. Subsequently we had to create scoring rules that reversed most of the items and labeled the scales as Not-Distracting and Not-Worrying. The items that created the original MAIA scales were selected from a large pool of items (originally over 100 collected in our focus groups, reduced to 63 for the original field test) according to factor analysis results. Although we viewed the need for reverse scoring as potentially problematic, we felt that we had to honor these earlier results when we created the two scales that showed poor Cronbach alphas in some samples. Therefore, although the reversing of items may potentially have contributed to the consistency issue, we decided to accept this as a minor evil and attempted to compensate by increasing the number of items.

### Analyses

We conducted exploratory and confirmatory factor analyses (CFA) and used the same statistical methods as described in detail in our original publication.[34] We created two separate subsamples by splitting the sample into two. We applied the SAS[35] PROC VARCLUS procedure to the observations with odd ID numbers including all 32+6 = 38 items as an equivalent for exploratory factor analysis (EFA). PROC VARCLUS begins with a principal components analysis of the correlation matrix and uses quartimax rotation for splitting, maximizing variances of loadings and accounting for the maximum amount of variance within the cluster. Although in the original MAIA we imputed a covariance matrix using the EM algorithm via SAS PROC MI as input to PROC VARCLUS, in the Science Museum sample only 12 of 1090 observations (7 in the odd ID subsample, 5 in the even ID subsample) had any missing data, so we dispensed with that step. We did not substitute missing data for incomplete observations and used only complete observations for the PROC VARCLUS analyses. Because the VARCLUS algorithm is a type of oblique component analysis, its output is similar to the output from the FACTOR procedure for oblique rotations. The cluster structure is analogous to the factor structure that contains the correlations between each variable and each cluster component. The methods applied in the analyses were part of an iterative decision process with potential elimination of items that performed relatively poorly during various steps of the analyses but keeping our 8-scale model.

For the final CFA we used observations with even ID numbers and Mplus Version7[36] with the remaining items. The maximum likelihood estimation method in Mplus included five participants with missing data. We allowed covariances between latent factors. We assessed fit indices and modification indices. Following conventional guidelines,[37] we required at least two[38] of the following fit indices to fall in the desired range: CFI > .90; RMSEA < .06; Tucker-Lewis index (TLI) > .95; standard root mean square residual (SRMR) < .08. Raw Cronbach’s alphas were assessed using the CORR procedure with SAS 9.4.[35] To compare independent Cronbach alpha values, we used the Feldt test.[39]

## Results

Our sample included 1090 participants and 12 had missing data. Forty-seven percent of the sample was female; the mean age was 30.6 years (SD = 11.3). Of note is that 60% of participants were native English speakers. We did not collect data about language fluency or level of acculturation for the non-native speakers. The split samples with odd and even IDs included 545 observations each. The VARCLUS procedure, our program analogue to an EFA, excluded 7 observations with missing values, whereas the Mplus program for the CFA did not exclude 5 incomplete observations. For this publication, we present the results of our factor analyses, item-scale correlations, scale-scale correlations and the Cronbach alphas for the eight scales in the study sample.

The VARCLUS procedure was performed on *n* = 538 observations (Table 1) excluding seven of 545 observations and generally confirmed the eight factors of the original MAIA. In addition, it suggested splitting one 5-item cluster corresponding to the Not-Worrying scale with a secondary Eigenvalue of 1.000045 into two scales of two and three items, respectively, thereby creating a ninth factor. We decided to dismiss this 9-cluster model as less parsimonious, as all 5 items were developed to capture elements for a single construct variable. Additionally, we wanted to maintain more than 3 items for this scale in order to improve its Cronbach’s alpha. One of the new added items—“*When I feel an unpleasant body sensation I just let it go by*”—did not load (for either native or nonnative English speakers) on the dimension to which it was hypothesized to belong and was deleted. The other five new items clustered with the factors as hypothesized. As we were concerned about the high proportion of non-native English speakers, we conducted sensitivity analysis in 329 native and 209 non-native English speakers, which confirmed the same 8-factor solution, and that the same item did not cluster with the intended factor.

**Table 1:** Cluster structure of the VARCLUS procedure in analogy to a factor structure of an exploratory factor analysis (all 38 items).

item	1	2	3	4	5	6	7	8
1	0.256	0.003	0.397	0.082	-0.060	0.308	0.179	**0.670**
2	0.194	-0.013	0.361	0.042	-0.163	0.249	0.116	**0.782**
3	0.253	0.019	0.285	0.214	-0.007	0.176	0.157	**0.721**
4	0272	0.074	0.295	0.101	-0.017	0.165	0.142	**0.633**
5	-0.040	**0.428**	-0.177	-0.023	0.192	-0.113	-0.096	-0.123
6	0.136	**0.534**	0.093	0.110	0.030	0.054	0.134	0.091
7	0.209	**0.616**	0.082	0.152	0.233	0.004	0.121	0.086
8	0.079	0.132	-0.186	0.094	**0.677**	-0.169	-0.018	-0.127
9	0.002	0.108	-0.257	0.047	**0.752**	-0.137	-0.004	-0.193
10	0.287	0.189	0.063	0.151	**0.519**	0.070	0.195	0.140
11	**0.598**	0.093	0.167	0.171	0.166	0.232	0.386	0.206
12	**0.737**	0.078	0.275	0.193	0.106	0.388	0.366	0.336
13	**0.639**	0.069	0.149	0.241	0.208	0.243	0.257	0.116
14	**0.789**	0.169	0.235	0.231	0.145	0.332	0.396	0.242
15	**0.709**	0.035	0.270	0.207	0.064	0.349	0.318	0.245
16	**0.719**	0.229	0.261	0.326	0.219	0.334	0.388	0.258
17	**0.725**	0.118	0.365	0.352	0.124	0.396	0.413	0.262
18	0.322	0.053	**0.693**	0.173	-0.082	0.404	0.275	0.339
19	0.176	-0.070	**0.663**	0.091	-0.198	0.425	0.178	0.332
20	0.258	0.085	**0.736**	0.134	-0.066	0.288	0.302	0.338
21	0.280	0.079	**0.761**	0.151	-0.141	0.306	0.343	0.363
22	0.264	0.045	**0.826**	0.207	-0.156	0.345	0.335	0.379
23	0.441	0.106	0.227	0.344	0.164	0.359	**0.691**	0.172
24	0.431	0.084	0.417	0.287	0.034	0.416	**0.782**	0.235
25	0.342	0.113	0.269	0.264	0.092	0.313	**0.791**	0.097
26	0.387	0.148	0.301	0.331	0.102	0.423	**0.835**	0.149
27	0.407	0.033	0.456	0.238	-0.095	**0.840**	0.418	0.306
28	0.355	-0.024	0.379	0.205	-0.103	**0.856**	0.429	0.244
29	0.407	0.047	0.355	0.304	-0.035	**0.814**	0.373	0.252
30	0.302	0.109	0.179	**0.882**	0.165	0.253	0.311	0.096
31	0.290	0.120	0.135	**0.917**	0.197	0.210	0.364	0.077
32	0.327	0.111	0.234	**0.809**	0.054	0.319	0.353	0.242
33	0.055	**0.735**	-0.047	0.040	0.266	-0.057	0.002	-0.035
34	0.135	**0.762**	0.139	0.146	0.123	0.088	0.203	0.028
35	0.091	**0.758**	0.133	0.089	0.031	0.049	0.118	0.070
36	0.131	***0*.*649***	-0.051	0.056	0.253	0.028	0.126	-0.031
37	0.270	0.361	0.018	0.224	**0.648**	0.080	0.263	0.005
38	0.099	0.028	-0.149	0.041	**0.663**	-0.108	0.013	-0.067

In the CFA of the final 37 items in the other half of the split sample (*n* = 545), two indices, namely RMSEA and SRMR, met our fit requirements: RMSEA = .055 (95% Confidence Interval .052 - .058; CFI = .860; TLI = .845; SRMR = .064; and Chi^2^ = 1597.7 (*df* 601, *p* < .0001).

In Table 2 we present factor loadings for the final 37 items, 32 original and 5 new items. The factor loading was lowest for the original item 5 (“I ignore physical tension or discomfort until they become more severe (R)”) with 0.30. All other items for this scale loaded at 0.52 or higher.

**Table 2:** Items and standardized CFA loadings for MAIA scales in CFA sample (*n* = 545).

	Standardized Loading	SE
**Noticing** 1. When I am tense, I notice where the tension is located in my body.	0.599	0.038
2. I notice when I am uncomfortable in my body.	0.640	0.038
3. I notice where in my body I am comfortable.	0.467	0.042
4. I notice changes in my breathing, such as whether it slows down or speeds up.	0.497	0.042
**Non-Distracting** 5. I ignore physical tension or discomfort until they become more severe.	0.296	0.044
6. I distract myself from sensations of discomfort.	0.523	0.037
7. When I feel pain or discomfort, I try to power through it.	0.573	0.036
8. I try to ignore pain	0.721	0.029
9. I push feelings of discomfort away by focusing on something	0.734	0.029
10. When I feel unpleasant body sensations, I occupy myself with something else so I don’t have to feel them.	0.682	0.030
**Not-Worrying** 11. When I feel physical pain, I become upset.	0.547	0.040
12. I start to worry that something is wrong if I feel any discomfort.	0.610	0.040
13. I can notice an unpleasant body sensation without worrying about it.	0.427	0.044
14. I can stay calm and not worry when I have feelings of discomfort or pain.	0.599	0.042
15. When I am in discomfort or pain I can’t get it out of my mind	0.521	0.042
**Attention Regulation** 16. I can pay attention to my breath without being distracted by things happening around me.	0.513	0.036
17. I can maintain awareness of my inner bodily sensations even when there is a lot going on around me.	0.612	0.031
18. When I am in conversation with someone, I can pay attention to my posture.	0.571	0.033
19. I can return awareness to my body if I am distracted.	0.715	0.026
20. I can refocus my attention from thinking to sensing my body.	0.733	0.025
21. I can maintain awareness of my whole body even when a part of me is in pain or discomfort.	0.623	0.031
22. I am able to consciously focus on my body as a whole.	0.681	0.028
**Emotional Awareness** 23. I notice how my body changes when I am angry.	0.506	0.036
24. When something is wrong in my life, I can feel it in my body.	0.628	0.031
25. I notice that my body feels different after a peaceful experience.	0.678	0.027
26. I notice that my breathing becomes free and easy, when I feel comfortable.	0.725	0.025
27. I notice how my body changes when I feel happy / joyful.	0.797	0.021
**Self-Regulation** 28. When I feel overwhelmed, I can find a calm place inside.	0.595	0.032
29. When I bring awareness to my body, I feel a sense of calm.	0.734	0.025
30. I can use my breath to reduce tension.	0.731	0.025
31. When I am caught up in thoughts, I can calm my mind by focusing on my body/breathing.	0.804	0.022
**Body Listening** 32. I listen for information from my body about my emotional state.	0.826	0.021
33. When I am upset, I take time to explore how my body feels.	0.759	0.024
34. I listen to my body to inform me about what to do.	0.723	0.026
**Trusting** 35. I am at home in my body.	0.822	0.020
36. I feel my body is a safe place.	0.889	0.019
37. I trust my body sensations.	0.669	0.028

Cronbach alphas were assessed in the complete sample *N* = 1090 for all eight scales and are presented in Table 3. Using all six items for Not-Distracting, three original and the three new items, the alpha was 0.74. Deleting the original item 5 (“I ignore physical tension or discomfort until they become more severe (R)”) would only marginally increase alpha to 0.76. This item had the lowest item-scale correlation of 0.39 with all other items correlating > 0.62. All six items are reverse scored.

**Table 3:** Basic descriptive statistics for the eight MAIA scales with Cronbach alphas, scale means and range of item-scale correlations.

	# of items	Item numbers	alpha MAIA-2	scale means (SD)	Range of item-scale correlations	alpha original MAIA[1]	alpha primary care patients[31]	Alpha German validation[21]
Noticing	4	1–4	.64	3.34 (0.90)	0.47–0.64	.69	.74	.76
Not-Distracting (3 new)	6	5–10	.74	2.06 (0.80)	0.30–0.73	.66	.48	.56
Not-Worrying (2 new)	5	11–15	.67	2.52 (0.85)	0.43–0.61	.67	.58	.65
Attention Regulation	7	16–22	.83	2.84 (0.86)	0.51–0.73	.87	.88	.89
Emotional Awareness	5	23–27	.79	3.44 (0.96)	0.51–0.80	.82	.90	.86
Self-Regulation	4	28–31	.79	2.78 (1.01)	0.60–0.80	.83	.86	.84
Body Listening	3	32–34	.80	2.20 (1.17)	0.72–0.83	.82	.83	.84
Trust	3	35–37	.83	3.37 (1.11)	0.67–0.89	.79	.78	.86

Cronbach alphas for the eight scales ranged from 0.64 to 0.83 (Table 3). Two were below the standard criterion of 0.70 –Noticing (.64) and Not Worrying (.67). All item-scale correlations met our criterion of 0.30, although the original item 5 did so barely.

Due to the high number of non-native English speakers, we conducted a sensitivity analysis for Cronbach’s alphas in the native and non-native subsamples, *n* = 650 and *n* = 440, respectively. For the first three scales, Cronbach alphas were maximally .05 different from the total sample, for the remaining five scales only .01 or .02. The largest difference between alphas for native and nonnative speakers was on Noticing, where Cronbach’s alpha for non-native speakers was actually higher than for native speakers (.69 vs. .60).

In order to compare the Cronbach alphas of the MAIA-2 in our sample with Cronbach alphas from the original development sample in practitioners of mind-body approaches,[1] the validation sample in primary care patients,[31] and a validation study in a German sample,[21] we included these in Tables 2 and 4. It shows that in our museum sample, where participants were most likely mind-body inexperienced, five of the unchanged 6 scales scored slightly lower than in the original development sample and both comparison studies, whereas the alpha for Not-Distracting was markedly improved, and the alpha for Not-Worrying was somewhat improved, but only compared to the two studies with inexperienced participants.

**Table 4:** Feldt test for comparing Cronbach alphas of MAIA-2 with those of independent samples (*W* statistic and *p* values).

	alpha MAIA-2	Compared to original MAIA[1]			Compared to primary care patients[31]			Compared to German validation[21]		
		*α*	*W*	*p*	*α*	*W*	*p*	*α*	*W*	*p*
*N*	1090	309			443			1076		
Not-Distracting (3 new)	.74	.66	.76	.001	.48	.50	.000	.56	.59	.000
Not-Worrying (2 new)	.67	.67	1.0	.493	.58	.79	.001	.65	.94	.167

Scale-scale correlations are presented in Table 5 and generally are in the expected direction. The strongest correlations reach 0.52 between Self-Regulation and Body-Listening, and between Emotional Awareness and Body-Listening.

**Table 5:** Scale-scale correlations (Pearson Correlation Coefficients; total sample; *N* = 1090).

	N	ND	NW	AR	EA	SR	BL	T
Noticing	1.00							
Not-Distracting	-0.01	1.00						
Not-Worrying	-0.09	-0.25	1.00					
Attention Regulation	0.37	-0.13	0.18	1.00				
Emotional Awareness	0.50	-0.02	-0.21	0.38	1.00			
Self-Regulation	0.27	-0.11	-0.11	0.50	0.45	1.00		
Body Listening	0.36	0.03	-0.13	0.43	0.52	0.52	1.00	
Trust	0.16	-0.05	0.16	0.37	0.21	0.37	0.29	1.00

## Discussion

In order to improve the MAIA, we conducted factor analyses for the original MAIA and three additional items for each of two scales, which had been found to be of limited internal consistency reliability in numerous applications. The opportunity for this study arose during an experiential project at the London Science Museum. Due to the relatively large sample size, we were able to randomly split the sample in half and conduct exploratory cluster analysis (equivalent to EFA) on one sample, and CFA on the other half.

The 8-factor structure was confirmed. New items improved the two scales’ Cronbach’s alphas with the exception of one new item that loaded more strongly on a different scale in the cluster analysis and was not included in the final CFA. The original item 5 (“I ignore physical tension or discomfort until they become more severe (R)”) performed the poorest according to its factor loading, item-scale correlation and contribution to Cronbach’s alpha. However, based on focus group participant feedback emphasizing the importance of this item,[33, 34] we retained it for the MAIA-2. The other six scales were not changed. Particularly when compared with mind-body *in*experienced samples, the two problem scales Not-Distracting and Not-Worrying were improved in internal consistency reliability for the MAIA-2. To ensure that the high proportion of non-native English speakers did not have a major influence on our results we conducted sensitivity analyses. This suggested that whether or not participants were native English language speakers had little systematic influence on the factorial structure of the scale. We were not surprised that the Cronbach alphas of most of the six unchanged scales were slightly below those of participants in the comparison samples, as participants in these were different: either mind-body trained, already highly motivated to do such training, or paying closer attention to interoceptive pain perception.

All questionnaires are limited by self-report, particularly, as in our case, if a questionnaire assesses parameters that are subject to learning and training in mind-body modalities. There is no objective measurement that can be used to validate the MAIA scales. The participants in this sample clearly differ from the mind body-experienced responders of the sample that was used for developing the original MAIA.[40] The validation sample with primary care patients of the original MAIA had confirmed the 8-factor structure with slightly different factor loadings similar to the current study.[31] A further limitation is the character of the study population. Visitors to a science museum are not characterized in great detail. However, they are expected to be mostly healthy individuals and represent the general population. And lastly, commonly used approximate fit indices for CFA are not uniformly accepted by the research community. However, as our a-priori criterion for model fit was met, we hope that this new MAIA version may be at least as useful for future studies as the old one.[41, 42]

In summary, we found that adding five new items to the original 32-item MAIA version created a 37-item MAIA-2 with improved psychometrics. Future studies should use the new version. The MAIA-2 is public domain and available on our website www.osher.ucsf.edu/maia. (S1 Questionnaire)

## Supporting information

S1 Questionnaire MAIA-2 questionnaire. (DOCX)
