# Supplementary material for: The Multidimensional Assessment of Interoceptive Awareness, Version 2 (MAIA-2)
Source: PLoS One. 2018 Dec 4;13(12):e0208034. doi: 10.1371/journal.pone.0208034 (PMC6279042; doi:10.1371/journal.pone.0208034)
Supplement: S1 Questionnaire — (DOCX) [file pone.0208034.s001.docx]

**Multidimensional Assessment of Interoceptive Awareness**

**Version 2**

**(MAIA-2)**

(2018)

Contact: Wolf E. Mehling, MD

Osher Center for Integrative Medicine

University of California, San Francisco

1545 Divisadero St., 4th floor

San Francisco, CA 94115

Phone: 01 (415) 353 9506

mehlingw@ocim.ucsf.edu

http://www.osher.ucsf.edu/maia/

**Multidimensional Assessment of Interoceptive Awareness (Version 2)**

**Permission and Copyright**

Although the MAIA survey is copyrighted, it is available without charge and no written permission is required for its use. This assumes agreement with the following as a consequence of using a MAIA survey:

- Please refer to the survey using its complete name – Multidimensional Assessment of Interoceptive Awareness - and provide the appropriate citation.
- Modifications may be made without our written permission. However, please clearly identify any modifications in any publications as having been made by the users. If you modify the survey, please let us know for our records.
- We recommend including entire subscales when selecting items from the MAIA to retain the psychometric features of these subscales (rather than selecting items from subscales).
- If you translate the MAIA into another language, please send us a copy for our records.
- If other investigators are interested in obtaining the survey, please refer them to the source document (PLoS-ONE 2012, and www.osher.ucsf.edu/maia/) to assure they obtain the most recent version and scoring instructions.

**Scoring Instructions**

Take the average of the items on each scale.

**Note: (R): reverse-score (5 – x) items 5, 6, 7, 8, 9 and 10 on Not-Distracting, and items 11, 12 and 15 on Not-Worrying.**

# Noticing: Awareness of uncomfortable, comfortable, and neutral body sensations

Q1______ + Q2______ + Q3______ + Q4______ / 4 = ___________

# Not-Distracting: Tendency not to ignore or distract oneself from sensations of pain or discomfort

Q5(**R**)____ + Q6(**R**)____+ Q7(**R**)____+ Q8(**R**)____+Q9(**R**)____+Q10(**R**) / 6 = ___________

# Not-Worrying: Tendency not to worry or experience emotional distress with sensations of pain or discomfort

Q11(**R**)______ + Q12(**R**)______ + Q13______ + Q14______ + Q15 (**R**) / 5 = ___________

# Attention Regulation: Ability to sustain and control attention to body sensations

Q16_____ + Q17_____ + Q18_____ + Q19_____ + Q20_____ + Q21_____ + Q22_____ / 7 = ________

1. **Emotional Awareness:** Awareness of the connection between body sensations and emotional states

Q23_____ + Q24_____ + Q25_____ + Q26_____ + Q27_____ / 5 = ___________

1. **Self-Regulation:** Ability to regulate distress by attention to body sensations

Q28_____ + Q29_____ + Q30_____ + Q31_____ / 4= ___________

1. **Body Listening:** Active listening to the body for insight

Q32_____ + Q33_____ + Q34_____ / 3= ___________

1. **Trusting:** Experience of one’s body as safe and trustworthy

Q35_____ + Q36_____ + Q37_____ / 3= ___________

**Below you will find a list of statements. Please indicate how often each statement applies to you generally in daily life.**

| . | **Circle one number on each line** | | | | | |
| --- | --- | --- | --- | --- | --- | --- |
|  | **Never** | |  |  | **Always** | |
| 1. When I am tense I notice where the tension is located in my body. | 0 | 1 | 2 | 3 | 4 | 5 |
| 2. I notice when I am uncomfortable in my body. | 0 | 1 | 2 | 3 | 4 | 5 |
| 3. I notice where in my body I am comfortable. | 0 | 1 | 2 | 3 | 4 | 5 |
| 4. I notice changes in my breathing, such as whether it slows down or speeds up. | 0 | 1 | 2 | 3 | 4 | 5 |
| 5. I ignore physical tension or discomfort until they become more severe. | 0 | 1 | 2 | 3 | 4 | 5 |
| 6. I distract myself from sensations of discomfort. | 0 | 1 | 2 | 3 | 4 | 5 |
| 7. When I feel pain or discomfort, I try to power through it. | 0 | 1 | 2 | 3 | 4 | 5 |
| 1. I try to ignore pain | 0 | 1 | 2 | 3 | 4 | 5 |
| 1. I push feelings of discomfort away by focusing on something | 0 | 1 | 2 | 3 | 4 | 5 |
| 1. When I feel unpleasant body sensations, I occupy myself with something else so I don’t have to feel them. | 0 | 1 | 2 | 3 | 4 | 5 |
| 11. When I feel physical pain, I become upset. | 0 | 1 | 2 | 3 | 4 | 5 |
| 12. I start to worry that something is wrong if I feel any discomfort. | 0 | 1 | 2 | 3 | 4 | 5 |
| 13. I can notice an unpleasant body sensation without worrying about it. | 0 | 1 | 2 | 3 | 4 | 5 |
| 14. I can stay calm and not worry when I have feelings of discomfort or pain. | 0 | 1 | 2 | 3 | 4 | 5 |
| 15. When I am in discomfort or pain I can’t get it out of my mind | 0 | 1 | 2 | 3 | 4 | 5 |
| 16. I can pay attention to my breath without being distracted by things happening around me. | 0 | 1 | 2 | 3 | 4 | 5 |
| 17. I can maintain awareness of my inner bodily sensations even when there is a lot going on around me. | 0 | 1 | 2 | 3 | 4 | 5 |
| 18. When I am in conversation with someone, I can pay attention to my posture. | 0 | 1 | 2 | 3 | 4 | 5 |

**How often does each statement apply to you generally in daily life? Circle one number on each line**

|  | **Never** |  |  | **Always** |  |  |
| --- | --- | --- | --- | --- | --- | --- |
| 19. I can return awareness to my body if I am distracted. | 0 | 1 | 2 | 3 | 4 | 5 |
| 20. I can refocus my attention from thinking to sensing my body. | 0 | 1 | 2 | 3 | 4 | 5 |
| 21. I can maintain awareness of my whole body even when a part of me is in pain or discomfort. | 0 | 1 | 2 | 3 | 4 | 5 |
| 22. I am able to consciously focus on my body as a whole. | 0 | 1 | 2 | 3 | 4 | 5 |
| 23. I notice how my body changes when I am angry. | 0 | 1 | 2 | 3 | 4 | 5 |
| 24. When something is wrong in my life I can feel it in my body. | 0 | 1 | 2 | 3 | 4 | 5 |
| 25. I notice that my body feels different after a peaceful experience. | 0 | 1 | 2 | 3 | 4 | 5 |
| 26. I notice that my breathing becomes free and easy when I feel comfortable. | 0 | 1 | 2 | 3 | 4 | 5 |
| 27. I notice how my body changes when I feel happy / joyful. | 0 | 1 | 2 | 3 | 4 | 5 |
| 28. When I feel overwhelmed I can find a calm place inside. | 0 | 1 | 2 | 3 | 4 | 5 |
| 29. When I bring awareness to my body I feel a sense of calm. | 0 | 1 | 2 | 3 | 4 | 5 |
| 30. I can use my breath to reduce tension. | 0 | 1 | 2 | 3 | 4 | 5 |
| 31. When I am caught up in thoughts, I can calm my mind by focusing on my body/breathing. | 0 | 1 | 2 | 3 | 4 | 5 |
| 32. I listen for information from my body about my emotional state. | 0 | 1 | 2 | 3 | 4 | 5 |
| 33. When I am upset, I take time to explore how my body feels. | 0 | 1 | 2 | 3 | 4 | 5 |
| 34. I listen to my body to inform me about what to do. | 0 | 1 | 2 | 3 | 4 | 5 |
| 35. I am at home in my body. | 0 | 1 | 2 | 3 | 4 | 5 |
| 36. I feel my body is a safe place. | 0 | 1 | 2 | 3 | 4 | 5 |
| 37. I trust my body sensations. | 0 | 1 | 2 | 3 | 4 | 5 |
